# Supplementary material for: Prognostic Clinical and Biologic Features for Overall Survival after Relapse in Childhood Medulloblastoma
Source: Cancers (Basel). 2020 Dec 27;13(1):53. doi: 10.3390/cancers13010053 (PMC7795432; doi:10.3390/cancers13010053)
Supplement: Supplementary file 1 [file cancers-13-00053-s001.zip › cancers-1047457-supplementary.docx]

**Table S1.** Main clinico-biological characteristics of patients with a relapsed medulloblastoma (n=48)

| Patient | Age - group at diagnosis | Sex | Histology | DNA-methylation Subgroup | M-stage (Chang's classification) | MYC-status | Initial treatment | RT doses  FP /  CSI/  Boost | Time between diagnosis and 1st relapse (years) | Site of relapse | Treatment at relapse | RT doses FP /  CSI | OS post-  relapse  (years) | Status |
| --- | --- | --- | --- | --- | --- | --- | --- | --- | --- | --- | --- | --- | --- | --- |
| 1 | < 5 | Female | Classic | N/A | M0 | No | CT alone |  | 0.13 | Local | CT alone |  | 0.49 | Dead |
| 2 | < 5 | Female | Nodular / Desmoplastic | SHH | M0 | No | CT alone |  | 1.17 | Local and metastatic | CT alone |  | 0.15 | Dead |
| 3 | < 5 | Male | Nodular / Desmoplastic | SHH | M0 | No | CT alone |  | 1.29 | Local | CT + HDCT + RT | 50.4 Gy (FP) | 6.46 | Dead |
| 4 | < 5 | Female | Nodular / Desmoplastic | SHH | M0 | No | CT alone |  | 1.75 | Local | SHH-inhib |  | 8.24 | ANED |
| 5 | < 5 | Male | Nodular / Desmoplastic | SHH | M0 | *MYCN* | CT alone |  | 0.51 | Local and metastatic | CT + HDCT + RT | 25.2/  50.4 Gy | 7.98 | ANED |
| 6 | < 5 | Male | Nodular / Desmoplastic | SHH | M0 | No | CT alone |  | 0.95 | Local | RT alone |  | 0.43 | Dead |
| 7 | < 5 | Male | Nodular / Desmoplastic | SHH | M0 | No | CT alone |  | 1.38 | Local and metastatic | CT + SHH-inhib | 54Gy (TB) | 0.98 | Dead |
| 8 | < 5 | Female | Nodular / Desmoplastic | SHH | M3 | No | CT alone |  | 0.94 | Metastatic | SHH-inhib |  | 2.00 | Dead |
| 9 | ≥ 5 | Male | Classic | G4 | M0 | No | CT alone |  | 1.50 | Local and metastatic | CT alone | NA | 0.40 | Dead |
| 10 | ≥ 5 | Female | Classic | G4 | M0 | No | CT alone |  | 1.76 | Local | RT alone | 36/  54 Gy | 10.00 | ANED |
| 11 | < 5 | Male | Classic | G4 | M0 | No | CT + HDCT |  | 2.07 | Local | CT + RT | 36/  54 Gy | 3.46 | ANED |
| 12 | < 5 | Male | Classic | NA | M3 | No | CT + HDCT |  | 2.02 | Local and metastatic | RT alone | 36/  54 Gy | 3.08 | ANED |
| 13 | < 5 | Male | Anaplastic / Large cell | G3 | M3 | No | CT + HDCT |  | 0.14 | Local and metastatic | CT alone |  | 0.27 | Dead |
| Patient | **Age - group at diagnosis** | **Sex** | **Histology** | **DNA-methylation Subgroup** | **M-stage (Chang's classification)** | **MYC-status** | **Initial treatment** | **RT doses**  **FP /**  **CSI/**  **Boost** | **Time between diagnosis and 1st relapse (years)** | **Site of relapse** | **Treatment at relapse** | **RT doses FP /**  **CSI** | **OS post-**  **relapse**  **(years)** | **Status** |
| 14 | ≥ 5 | Male | Classic | G4 | M0 | No | RT alone | 36/60/68 Gy  (CSI/FP/TB) | 3.50 | Metastatic | CT alone |  | 0.70 | Dead |
| 15 | ≥ 5 | Male | NOS | WNT | M0 | No | RT alone | 36/68 Gy | 1.73 | Metastatic | CT alone |  | 5.57 | ANED |
| 16 | ≥ 5 | Female | Classic | G4 | M0 | No | RT alone | 36/68 Gy | 2.08 | Metastatic | CT alone |  | 0.66 | ANED |
| 17 | ≥ 5 | Male | Classic | WNT | M0 | No | RT alone | 36/68 Gy | 1.33 | Metastatic | CT alone |  | 2.05 | Dead |
| 18 | ≥ 5 | Male | Classic | NA | M0 | No | CT+RT | 36/68 Gy | 3.95 | Metastatic | CT alone |  | 0.50 | ANED |
| 19 | ≥ 5 | Male | Classic | G4 | M0 | No | CT+RT | 36/68 Gy | 2.49 | Metastatic | CT alone |  | 2.27 | Dead |
| 20 | ≥ 5 | Female | Anaplastic / Large cell | G4 | M0 | MYCN | CT+RT | 36/68 Gy | 1.60 | Local | RT alone | 24 Gy (TB) | 1.10 | Dead |
| 21 | < 5 | Female | Anaplastic / Large cell | G3 | M2 | Yes | CT + HDCT + RT | NA | 0.10 | Local and metastatic | CT+HDCT |  | 0.32 | Dead |
| 22 | < 5 | Male | Classic | NA | M2 | *MYCN* | CT + HDCT + RT | NA | 0.14 | Local and metastatic | None |  | 0.05 | Dead |
| 23 | < 5 | Male | Classic | G3 | M0 | No | CT + HDCT + RT | 50.4 Gy (TB) | 1.08 | Local and metastatic | CT alone |  | 0.07 | Dead |
| 24 | < 5 | Female | Classic | G4 | M0 | No | CT + HDCT + RT | 36/50.4 Gy (FP/TB) | 4.35 | Local and metastatic | CT alone |  | 3.34 | Dead |
| 25 | < 5 | Female | Classic | G3 | M0 | No | CT + HDCT + RT | 43.2/49 Gy (FP/TB) | 0.90 | Metastatic | CT alone |  | 0.31 | Dead |
| 26 | < 5 | Female | Classic | G3 | M0 | No | CT + HDCT + RT | 40 Gy (TB) | 1.03 | Local and metastatic | CT alone |  | 1.47 | Dead |

| Patient | Age - group at diagnosis | Sex | Histology | DNA-methylation Subgroup | M-stage (Chang's classification) | MYC-status | Initial treatment | RT doses  FP /  CSI/  Boost | Time between diagnosis and 1st relapse (years) | Site of relapse | Treatment at relapse | RT doses FP /  CSI | OS post-  relapse  (years) | Status |
| --- | --- | --- | --- | --- | --- | --- | --- | --- | --- | --- | --- | --- | --- | --- |
| 27 | < 5 | Female | Classic | G3 | M0 | No | CT + HDCT + RT | 50.4 Gy (FP) | 0.89 | Local and metastatic | CT alone |  | 0.17 | Dead |
| 28 | < 5 | Female | Anaplastic / Large cell | G4 | M0 | *MYC* | CT + HDCT + RT | 25.2/54 Gy | 0.84 | Local and metastatic | CT alone |  | 0.13 | Dead |
| 29 | < 5 | Male | Classic | G3 | M3 | No | CT + HDCT + RT | NA | 0.14 | Local and metastatic | CT alone |  | 0.12 | Dead |
| 30 | < 5 | Female | Anaplastic / Large cell | G3 | M1 | No | CT + HDCT + RT | NA | 0.45 | Local and metastatic | CT alone |  | 0.22 | Dead |
| 31 | < 5 | Female | Classic | G3 | M0 | No | CT + HDCT + RT | NA | 0.37 | Local and metastatic | CT alone |  | 0.19 | Dead |
| 32 | < 5 | Male | Anaplastic / Large cell | G3 | M3 | MYC | CT + HDCT + RT | NA | 0.24 | Metastatic | CT alone |  | 0.07 | Dead |
| 33 | < 5 | Male | NOS | G3 | M3 | No | CT + HDCT + RT | 36/54 /9 Gy | 1.07 | Metastatic | CT alone |  | 0.28 | Dead |
| 34 | < 5 | Male | Classic | NA | M3 | No | CT + HDCT + RT | NA | 0.18 | Metastatic | CT alone |  | 0.01 | Dead |
| 35 | ≥ 5 | Female | Classic | G4 | M2 | *MYCN* | CT + HDCT + RT | 36/54 Gy | 4.01 | Local | CT alone |  | 2.59 | Dead |
| 36 | ≥ 5 | Male | Nodular / Desmoplastic | SHH | M3 | No | CT + HDCT + RT | 36/54 Gy | 7.27 | Metastatic | None |  | 1.49 | Dead |
| 37 | ≥ 5 | Female | Classic | G3 | M3 | No | CT + HDCT + RT | NA | 0.16 | Local and metastatic | None |  | 0.04 | Dead |
| 38 | ≥ 5 | Female | Classic | G3 | M3 | No | CT + HDCT + RT | 36/54 Gy | 0.67 | Metastatic | None |  | 0.02 | Dead |
| 39 | ≥ 5 | Male | Anaplastic / Large cell | G3 | M3 | MYC | CT + HDCT + RT | 36/54 Gy | 0.68 | Metastatic | CT alone |  | 0.14 | Dead |
| 40 | ≥ 5 | Male | NOS | G3 | M3 | No | CT + HDCT + RT | 36/54 Gy | 2.06 | Metastatic | CT alone |  | 0.91 | Dead |
| Patient | **Age - group at diagnosis** | **Sex** | **Histology** | **DNA-methylation Subgroup** | **M-stage (Chang's classification)** | **MYC-status** | **Initial treatment** | **RT doses**  **FP /**  **CSI/**  **Boost** | **Time between diagnosis and 1st relapse (years)** | **Site of relapse** | **Treatment at relapse** | **RT doses FP /**  **CSI** | **OS post-**  **relapse**  **(years)** | **Status** |
| 41 | ≥ 5 | Female | Classic | G4 | M3 | MYC | CT + HDCT + RT | 39.6/54 Gy | 4.06 | Metastatic | CT alone |  | 2.68 | Dead |
| 42 | ≥ 5 | Female | Classic | NA | M3 | No | CT + HDCT + RT | 36/54 Gy | 1.74 | Local | RT alone | 6 Gy (TB) | 0.99 | Dead |
| 43 | ≥ 5 | Male | Classic | G3 | M2 | No | CT + HDCT + RT | 36/54 Gy | 1.21 | Metastatic | SHH-inhib |  | 0.15 | Dead |
| 44 | ≥ 5 | Male | Nodular / Desmoplastic | SHH | M3 | No | CT + HDCT + RT | 36/54/  5.6 Gy | 2.68 | Metastatic | SHH-inhib |  | 3.13 | AWD |
| 45 | ≥ 5 | Male | Classic | G3 | M3 | No | CT + HDCT + RT | 36/54 Gy | 0.66 | Metastatic | CT alone |  | 0.33 | Dead |
| 46 | ≥ 5 | Female | Classic | G4 | M2 | No | CT + HDCT + RT | 36/54/18 Gy | 2.64 | Metastatic | CT alone |  | 1.11 | ANED |
| 47 | ≥ 5 | Male | Classic | G3 | M3 | No | CT + HDCT + RT | NA | 0.34 | Metastatic | CT + RT | 36/  54 Gy | 1.22 | Dead |
| 48 | ≥ 5 | Male | Classic | G4 | M2 | No | CT + HDCT + RT | 36/54 Gy | 1.43 | Metastatic | CT alone |  | 0.82 | AWD |

G3: Group 3; G4: Group 4; CT: chemotherapy; HDCT: High dose CT; RT: radiotherapy; FP: fossa posterior; TB: tumor bed; AWD: Alive without disease; ANED: Alive no evidence of disease

| Patient | Age at onset | Histology | Subgroup affiliation (DNA -methylation) | *MYC/MYCN* amplification  status | M-stage | Initial treatment | Doses of RT at  diagnosis  CSI/  PF TB | Time  between  diagnosis  and  relapse (years) | Site of relapse | Treatment at relapse | Doses of RT at relapse  CSI/  PF TB | Further event | Status | | Survival after relapse  (years) |
| --- | --- | --- | --- | --- | --- | --- | --- | --- | --- | --- | --- | --- | --- | --- | --- |
| 1 | 7.3 | Classic | Group 4 | No | M0 | CT alone |  | 1.76 | local | RT alone | 36 Gy/  54 Gy |  | | ANED | 10 |
| 2 | 3.1 | Desmoplastic/  nodular | SHH | No | M0 | CT alone |  | 1.75 | local | SHH-inhib |  |  | | ANED | 8.24 |
| 3 | 8.8 | Classic | *NA* | No | M0 | RT-CT | 36Gy/  54Gy | 3.95 | metastatic | CT |  |  | | ANED | 0.5 |
| 4 | 3.3 | Desmoplastic/  nodular | SHH | *MYCN* | M0 | CT alone |  | 0.51 | local and metastatic | CT-HDCT- RT | 25.5Gy/  50.4Gy |  | | ANED | 7.98 |
| 5 | 9.9 | NOS | WNT | No | M0 | RT alone | 36Gy/  68Gy | 1.73 | metastatic | CT |  |  | | ANED | 5.57 |
| 6 | 9.3 | Classic | Group 4 | No | M2 | CT-HDCT- RT |  | 1.43 | metastatic | CT |  |  | | AWD | 0.82 |
| 7 | 11.8 | Classic | Group 4 | No | M0 | RT alone | 36Gy/  68Gy | 2.08 | metastatic | CT |  |  | | ANED | 0.66 |
| 8 | 13.9 | Desmoplastic/  nodular | SHH | No | M3 | CT-HDCT- RT | 36Gy/  54Gy/  Boost M+ 5Gy | 2.68 | metastatic | SHH-inhib |  | Further progression, treated with SHH-inhib – Temodal | | AWD | 3.13 |
| 9 | 2.7 | Classic | Group 4 | No | M0 | CT-HDCT |  | 2.07 | local | CT-RT | 36Gy/  54Gy |  | | ANED | 3.46 |
| 10 | 4.0 | Classic | *NA* | No | M3 | CT-HDCT |  | 2.02 | local and metastatic | RT alone | 36Gy/  54Gy |  | | ANED | 3.08 |
| 11 | 10.3 | Classic | Group 4 | No | M2 | CT-HDCT-RT | 36Gy/  54Gy/  Boost M+ 18Gy | 2.64 | metastatic | CT |  |  | | ANED | 1.11 |

**Table S2.** Clinical and biological data, treatment at diagnosis and at relapse, site of relapse and outcome among 11 out 48 relapsed patients alive at last follow-up

Abbreviations: NA: not available; CT: chemotherapy; RT: craniospinal radiotherapy; CSI: craniospinal; PF TB: posterior fossa tumor bed; HDCT: High dose chemotherapy; M+: metastases; SHH-inhib: Sonic Hedgehog-inhibitor; ANED: Alive no evidence of disease; AWD: Alive with disease.
